# Association of visceral and subcutaneous adiposity with tumor stage and Fuhrman grade in renal cell carcinoma

**DOI:** 10.1038/s41598-022-20877-2

**Published:** 2022-10-06

**Authors:** J. S. F. Maurits, J. P. M. Sedelaar, K. K. H. Aben, L. A. L. M. Kiemeney, A. Vrieling

**Affiliations:** 1 Department for Health Evidence (Mailbox 133), Radboud University Medical Center, P.O. Box 9101, 6500 HB Nijmegen, The Netherlands; 2 Department of Urology, Radboud University Medical Center, Nijmegen, The Netherlands; 3 Department of Research and Development, Netherlands Comprehensive Cancer Organisation, Utrecht, The Netherlands

**Keywords:** Renal cancer, Tumour biomarkers, Urological cancer

## Abstract

Higher BMI has been associated with lower tumor stage and grade and improved survival in renal cell cancer (RCC). BMI cannot distinguish between visceral adipose tissue (VAT) and subcutaneous adipose tissue (SAT). We examined associations of BMI, VAT, SAT, total adipose tissue (TAT) and relative VAT (rVAT) with tumor stage and grade in RCC patients. In a Dutch multicenter population-based historical cohort study 1039 RCC patients diagnosed between 2008 and 2012 were assessed for VAT and SAT using Computed Tomography images at L3. Sex-stratified multinomial logistic regression analyses were performed (linearly per 10-unit increase) between BMI, VAT, SAT, TAT and relative VAT (rVAT) with tumor stage and Fuhrman grade. Higher VAT, TAT and rVAT were associated with a lower risk of stage IV versus stage I in males (OR 0.93; 95%CI 0.91–0.96, OR 0.95; 95%CI 0.93–0.98, OR 0.97; 95%CI 0.96–0.99, respectively). Females showed similar associations, but only higher VAT was statistically significantly associated with reduced risk of stage IV (OR 0.95 95%CI 0.89–1.00). No associations with grade, SAT or BMI were found. In conclusion, higher VAT and TAT was associated with lower risk of stage IV RCC. This might be due to weight loss or cancer cachexia in stage IV patients.

## Introduction

Body Mass Index (BMI) is a major risk factor for renal cell cancer (RCC)^1^ but high BMI has been associated with better recurrence-free and cancer-specific survival^2,3^. One possible explanation is that obese patients are more likely to be diagnosed with renal incidentalomas with low tumor stage and grade due to more frequent imaging for other diseases^4^. Another hypothesis is that patients with a lower BMI at diagnosis may have experienced weight loss before diagnosis due to a more aggressive disease. Indeed, some studies in patients with RCC found that higher BMI was related to lower tumor stage^4,5^ and lower Fuhrman grade^4–6^ while others found no associations^3,7–10^. On a molecular level, fatty acid synthase (FASN) expression, which is related to aggressive disease in RCC, seems to be downregulated in obese patients. This could also explain the longer survival in patients with higher BMI^11,12^. BMI cannot distinguish between visceral adipose tissue (VAT) and subcutaneous adipose tissue (SAT). Increased VAT has been shown to promote tumorigenesis^13^. Examining the association of body composition with tumor characteristics might provide more insight in the paradoxical association between BMI and survival in RCC.

Several studies have previously investigated the relationship between VAT and tumor stage and grade and reported inconsistent results. Two studies reported higher VAT in patients with higher tumor stage^8,14^ while two other studies found higher VAT in patients with low stage disease^9,15^. Several studies showed that higher VAT was associated with higher Fuhrman grade^7,10,14,16,17^, while only one study found that higher VAT was associated with lower International Society of Urologic Pathologists (ISUP) grade^18^. Three studies in localized RCC patients reported no associations between VAT and tumor stage^19–21^ or grade^19,21^.

It is known that males generally have higher VAT and lower SAT than females^22^ resulting in potential sex-specific metabolic risks^13^, which advocates sex-specific analyses. In RCC, one study reported that a higher ratio of VAT compared to total adipose tissue (TAT), termed relative VAT (rVAT), was associated with higher stage in both males and females^8^, while another study showed higher rVAT was associated with higher grade in females only^16^.

In this large population-based historical cohort study, we examined whether sex-specific BMI, VAT, SAT, TAT and relative VAT are related to tumor stage and Fuhrman grade in patients with RCC.

## Methods

### Study population

A population-based historical cohort study was conducted in 7 hospitals in the Netherlands: Radboud university medical center, Nijmegen; Amphia Hospital, Breda; Catharina Hospital, Eindhoven; Meander Medical Centre, Amersfoort; Rijnstate Hospital, Arnhem; Slingeland Hospital, Doetinchem; Ziekenhuisgroep Twente, Almelo and Hengelo.

The Committee for Human Research region Arnhem-Nijmegen (CMO 2015-1822) approved the study protocol and waived the need for informed consent. All local ethics committees from the participating hospitals provided permission for data collection. This study was conducted in compliance with the principles of the Declaration of Helsinki. This cohort study was also set up to examine body composition in relation to survival outcomes and has been described elsewhere^23^.

**Figure 1:**
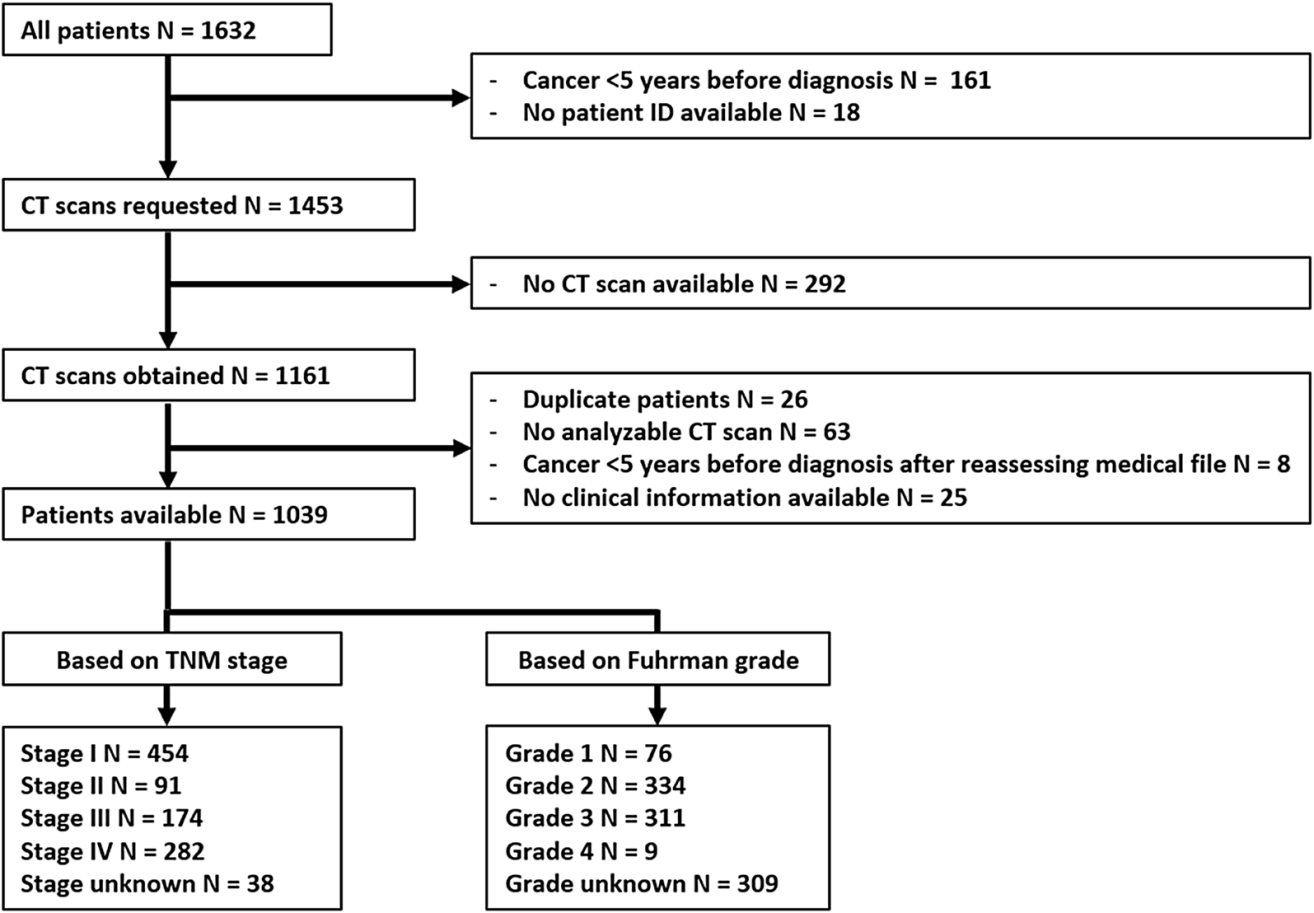
Flowchart of the included patients, separated by TNM stage and Fuhrman grade.

Urologists and radiologists from the 7 hospitals were asked for permission to identify RCC patients diagnosed in their hospital from the Netherlands Cancer Registry (NCR), held by the Netherlands Comprehensive Cancer Organisation (IKNL). Permission was also asked for collecting CT images obtained within 3 months before RCC diagnosis, and for retrospectively collecting information on body weight and height at RCC diagnosis and clinical data from the medical records by IKNL personnel. We applied the following inclusion criteria: First established primary stage I-IV RCC, diagnosed between 2008 and 2012, and 18 years of age or older at diagnosis. RCC patients were excluded if they had been diagnosed with cancer in the 5 years preceding RCC diagnosis. Patients without patient number, duplicate patients, and those without a CT scan or without an analyzable CT scan (due to too much graining/low contrasts or artefacts) were excluded (see Fig. 1).

### Body composition analysis

A single CT image at the level of the third lumbar vertebra (L3) was used to examine body composition parameters. It has been shown that the cross-sectional area of muscle and fat tissue measured at a single axial L3 slice is strongly correlated with the amount of muscle and fat tissue in the whole body^24^. Body composition parameters were quantified using Slice-O-Matic 5.0 software (TomoVision), based on commonly used density thresholds in Hounsfield units (HU); 29 to + 150 for Skeletal Muscle (SM), − 190 to − 30 for SAT and intramuscular adipose tissue (IMAT), and − 150 to − 50 for VAT^24,25^. Total cross-sectional areas were measured in cm^2^ and the cross-sectional area of SM was normalized for height (m^2^) to obtain the skeletal muscle index (SMI). We did not include IMAT in the statistical analysis. In case of incomplete cross-sectional areas of SAT (e.g. abdomen not fully visible due to poor positioning or large body size of the patient), the cut off SAT areas were estimated using an algorithm in MATLAB under the assumption that SAT is symmetrical. In this algorithm, the cut off SAT areas were estimated by extrapolating the contour of the abdomen to the cut off sections to create a complete abdomen. In total 313 out of the 1039 patients (30.1%) had adjusted SAT estimates using this algorithm. Total adipose tissue (TAT) was calculated by summing up VAT and SAT of a participant (TAT = VAT + SAT). rVAT was calculated by the ratio of VAT compared to TAT expressed as a percentage (rVAT = 100% * VAT/(VAT + SAT)). All CT scan analyses were performed by one trained researcher (J.S.F.M.) following the Alberta Protocol for uniform segmentation of a single CT slice at L3^25^. A small subset (n = 30) was checked by a second trained external researcher who was not part of the study to assess interrater reproducibility. The intraclass correlation coefficients were 0.992 for SMI, 0.999 for VAT and 0.979 for SAT.

### Clinical data collection

Data on tumor characteristics (clinical and post-surgical TNM stage, Fuhrman grade, morphology) were derived from the NCR. Additional preoperative information (age, gender, body weight, height) were extracted from medical records by IKNL data managers and stored into standardized electronic case forms in CASTOR EDC.

### Outcomes

The outcomes were TNM stage based on pathological completed with clinical TNM classification used in the year of incidence (6th edition up to and including 2009, 7th edition from 2010 to 2012) and Fuhrman grade of the tumor. Fuhrman grade 3 and 4 were combined due to low numbers of grade 4.

### Statistical analysis

Study sample characteristics were presented per outcome category (stage and grade). Descriptive characteristics included means and standard deviations (SD), medians and interquartile ranges (IQR), or total numbers and percentages (%), where appropriate.

Different adipose tissue components were presented per outcome in violin plots for males and females separately. The medians were tested for statistical differences with Kruskal–Wallis and Wilcoxon rank sum tests, given the skewed distributions of the components.

Univariable and multivariable multinomial logistic regression analyses were performed to assess the association of adipose tissue parameters with stage (stage I as referent category) and Fuhrman grade (grade 1 as referent category) using odds ratios (OR) and 95% confidence intervals (95%CI).

We first tested for non-linearity of the associations by examining 10-unit increases in body composition parameters. Associations for VAT, TAT and rVAT were linear in both males and females. The association of SAT with Fuhrman grade was non-linear and SAT was therefore also examined using sex-specific medians (dichotomous model). Multivariable regression analyses were adjusted for age and BMI and mutually adjusted for other adipose tissue components (i.e. SAT for VAT and vice versa). Multiple imputation was used to deal with missing data.

We performed two sensitivity analyses. First, since not all obtained CT scans were taken within 3 months before RCC diagnosis (N = 179, 17.2%), we performed a sensitivity analysis using only CT scans taken within 3 months before RCC diagnosis (N = 860, 82.8%).

Second, since clear cell RCC (ccRCC) is the subtype that is more strongly associated with obesity than other histological subtypes^26^, we performed a sensitivity analysis in patients with confirmed ccRCC only (N = 655, 63.0%).

All analyses were executed in R for Windows version 3.6.2 (i.e. packages “MICE”, “ggplot2”, “ggpubr”).

## Results

### Study sample characteristics

In total, 1039 patients were included (Fig. 1). Mean age was 64.8 ± 11.8 years and 643 patients (61.9%) were male. Of all patients, 43.7% had stage I, 8.8% stage II, 16.7% stage III and 27.1% stage IV disease while for 3.6% stage was unknown (Table 1). In total, 7.3% had Fuhrman grade 1, 32.1% had grade 2, 29.9% had grade 3 and 0.9% had grade 4, while for 29.8% grade was unknown (Fig. 1 and Table 1). Supplementary Figs. 1–4 present violin plots for adipose tissue parameters in males and females, separately. Statistically significant differences in VAT, SAT, TAT and rVAT were seen between stages and between grades within males and females, respectively, but no differences in rVAT between stages in females were found.

**Table 1:** Basic characteristics and body adipose tissues per TNM stage^a^ and Fuhrman grade.

Variable	Total (N = 1039)	Stage I (N = 454, 43.7%)	Stage II (N = 91, 8.8%)	Stage III (N = 174, 16.7%)	Stage IV (N = 282, 27.1%)	Unknown stage (N = 38, 3.7%)
Age at diagnosis (years), mean (SD)	64.8 (11.8)	63.5 (12.0)	62.9 (12.6)	66.0 (10.4)	65.1 (11.5)	76.4 (10.4)
**Sex**						
Male, n (%)	643 (61.9%)	267 (58.8%)	54 (59.3%)	118 (67.8%)	181 (64.2%)	23 (60.5%)
Female, n (%)	396 (38.1%)	187 (41.2%)	37 (40.7%)	56 (32.2%)	101 (35.8%)	15 (39.5%)
**Morphology**						
Confirmed ccRCC, n (%)	655 (63.0%)	295 (65.0%)	57 (62.6%)	134 (77.0%)	167 (59.2%)	2 (5.3%)
Non-ccRCC or not specified, n (%)	384 (37.0%)	159 (35.0%)	34 (37.4%)	40 (23.0%)	115 (40.8%)	36 (94.7%)
**BMI**						
Mean (SD)	26.6 (4.35)	27.1 (4.72)	27.0 (4.67)	26.2 (3.58)	25.8 (3.94)	26.7 (3.95)
Missing, n (%)	160 (15.4)	55 (12.1%)	7 (7.7%)	11 (6.3%)	76 (27.0%)	11 (28.9%)
VAT (cm^2^), median (IQR)	145 (73, 220)	168 (93, 239)	147 (59, 224)	149 (77, 214)	110 (53, 181)	135 (77, 244)
SAT (cm^2^), median (IQR)	156 (107, 218)	166 (123, 230)	167 (107, 213)	145 (105, 216)	137 (98, 189)	139 (97, 188)
TAT (cm^2^), median (IQR)	315 (216, 427)	346 (255, 452)	301 (211, 429)	326 (211, 399)	258 (176, 375)	302 (198, 441)
rVAT (%), median (IQR)	45.8 (30.7, 58.7)	46.4 (31.9, 60.1)	43.7 (30.1, 57.9)	48.7 (32.7, 60.7)	42.4 (28.5, 53.2)	53.2 (34.5, 62.3)

Variable	Total (N = 1039)	Grade 1 (N = 76, 7.3%)	Grade 2 (N = 334, 32.1%)	Grade (3 + 4) (N = 320, 30.8%)	Unknown grade (N = 309, 29.8%)	
Age at diagnosis, year mean (SD)	64.8 (11.8)	62.8 (11.6)	62.9 (11.7)	63.4 (11.2)	68.9 (11.8)	
**Sex**						
Male, n (%)	643 (61.9%)	34 (44.7%)	207 (62.0%)	220 (68.8%)	182 (58.9%)	
Female, n (%)	396 (38.1%)	42 (55.3%)	121 (38.0%)	100 (31.3%)	127 (41.1%)	
**Morphology**						
Confirmed ccRCC, n (%)	655 (63.0%)	57 (75.0%)	260 (77.8%)	236 (73.8%)	207 (67.0%)	
Non-ccRCC or not specified, n (%)	384 (37.0%)	19 (25.0%)	74 (22.2%)	84 (26.3%)	102 (33.0%)	
**BMI**						
Mean (SD)	26.6 (4.35)	27.9 (5.24)	26.9 (4.26)	26.2 (4.50)	26.3 (3.90)	
Missing, n (%)	160 (15.4)	9 (11.8%)	41 (12.3%)	39 (12.2%)	71 (23.0%)	
VAT (cm^2^), median (IQR)	145 (73, 220)	169 (111, 235)	159 (91, 237)	143 (62, 205)	125 (61, 204)	
SAT (cm^2^), median (IQR)	156 (107, 218)	199 (138, 241)	167 (120, 227)	140 (102, 191)	148 (99, 215)	
TAT (cm^2^), median (IQR)	315 (216, 427)	348 (290, 492)	345 (242, 447)	293 (186, 397)	288 (196, 401)	
rVAT (%), median (IQR)	45.8 (30.7, 58.7)	43.0 (31.3, 57.1)	46.4 (32.1, 59.5)	46.5 (31.2, 58.2)	44.6 (29.3, 59.7)	

ccRCC, clear cell renal cell carcinoma; BMI, body mass index; VAT, visceral adipose tissue; SAT, subcutaneous adipose tissue; TAT, total adipose tissue; rVAT, relative visceral adipose tissue.

^a^Tumor staging based on pathological completed with clinical TNM classification used in the year of incidence (6th edition up to and including 2009, 7th edition from 2010–2012).

### Adipose tissue and tumor stage

In univariable analyses, compared to stage I, higher BMI, VAT and TAT were statistically significantly associated with a reduced risk of stage III and stage IV, and higher SAT with a reduced risk of stage IV in both males and females. Higher rVAT was associated with reduced risk of stage IV in males only (Table 2).

**Table 2:** Odds ratios for body adipose tissue and TNM stage (stage I as referent) and Fuhrman grade (grade 1 as referent) for males and females separately.

TNM stage Stage I is referent	Univariable analysis			Multivariable analysis		
	Stage II OR (95% CI)	Stage III OR (95% CI)	Stage IV OR (95% CI)	Stage II OR (95% CI)	Stage III OR (95% CI)	Stage IV OR (95% CI)
**Males**						
BMI^a^, kg/m^2^	1.02 (0.95–1.09)	0.93 (0.87–0.98)*	0.90 (0.86–0.95)***	1.09 (0.95–1.25)	0.97 (0.87–1.08)	1.03 (0.93–1.14)
VAT^a^, per 10 cm^2^	0.99 (0.96–1.01)	0.97 (0.95–0.99)*	0.94 (0.92–0.96)***	0.97 (0.93–1.00)	0.98 (0.95–1.01)	0.93 (0.91–0.96)***
SAT^a^, per 10 cm^2^	1.01 (0.97–1.04)	0.97 (0.94–1.00)	0.96 (0.94–0.99)*	0.99 (0.94–1.05)	1.00 (0.96–1.05)	1.01 (0.96–1.05)
TAT^b^, per 10 cm^2^	1.00 (0.98–1.01)	0.98 (0.97–1.00)*	0.97 (0.95–0.98)***	0.97 (0.94–1.01)	0.98 (0.96–1.01)	0.95 (0.93–0.98)***
rVAT^b^, in %	0.99 (0.97–1.01)	0.99 (0.98–1.01)	0.97 (0.96–0.98)***	0.99 (0.97–1.01)	0.99 (0.98–1.01)	0.97 (0.96–0.99)***
**Females**						
BMI^a^, kg/m^2^	0.96 (0.88–1.03)	0.96 (0.90–1.03)	0.91 (0.86–0.97)**	0.99 (0.85–1.15)	1.01 (0.89–1.15)	0.99 (0.88–1.11)
VAT^a^, per 10 cm^2^	0.97 (0.93–1.02)	0.98 (0.94–1.02)	0.95 (0.92–0.99)**	0.98 (0.91–1.06)	0.96 (0.90–1.02)	0.95 (0.89–1.00)*
SAT^a^, per 10 cm^2^	0.98 (0.94–1.01)	0.98 (0.95–1.01)	0.96 (0.93–0.98)**	0.99 (0.93–1.05)	1.00 (0.94–1.05)	0.99 (0.94–1.03)
TAT^b^, per 10 cm^2^	0.99 (0.96–1.01)	0.99 (0.97–1.01)	0.97 (0.96–0.99)***	0.99 (0.94–1.03)	0.98 (0.94–1.02)	0.97 (0.94–1.00)
rVAT^b^, in %	0.99 (0.97–1.02)	1.00 (0.97–1.02)	0.99 (0.97–1.01)	0.99 (0.96–1.02)	0.99 (0.96–1.01)	0.98 (0.96–1.00)

Fuhrman grade Grade 1 is referent	Univariable analysis			Multivariable analysis		
	Grade 2 OR (95% CI)	Grade 3 + 4 OR (95% CI)		Grade 2 OR (95% CI)	Grade 3 + 4 OR (95% CI)	
**Males**						
BMI^a^, kg/m^2^	1.03 (0.98–1.08)	0.99 (0.94–1.04)		0.96 (0.88–1.06)	0.98 (0.89–1.08)	
VAT^a^, per 10 cm^2^	1.01 (1.00–1.03)	0.99 (0.97–1.00)		1.03 (1.00–1.05)	0.99 (0.96–1.02)	
SAT^a^, per 10 cm^2^	1.02 (0.99–1.05)	1.01 (0.98–1.03)		1.01 (0.97–1.05)	1.02 (0.98–1.06)	
SAT^a^, high vs low	1.51 (1.03–2.22)*	0.95 (0.65–1.38)		1.14 (0.72–1.82)	0.94 (0.60–1.47)	
TAT^b^, per 10 cm^2^	1.01 (1.00–1.02)	0.99 (0.98–1.01)		1.02 (1.00–1.05)	1.00 (0.98–1.02)	
rVAT^b^, in %	1.00 (0.99–1.01)	0.99 (0.97–1.00)		1.01 (0.99–1.02)	0.99 (0.94–1.05)	
**Females**						
BMI^a^, kg/m^2^	1.02 (0.97–1.07)	0.98 (0.93–1.03)		0.97 (0.88–1.07)	1.02 (0.92–1.14)	
VAT^a^, per 10 cm^2^	1.01 (0.98–1.04)	0.97 (0.94–1.01)		1.03 (0.98–1.08)	1.00 (0.95–1.05)	
SAT^a^, per 10 cm^2^	1.01 (0.99–1.04)	0.99 (0.96–1.01)		1.01 (0.97–1.05)	0.98 (0.93–1.02)	
SAT^a^, high vs low	1.39 (0.87–2.22)	0.59 (0.36–0.98)*		1.10 (0.59–2.06)	0.51 (0.26–0.99)*	
TAT^b^, per 10 cm^2^	1.01 (0.99–1.02)	0.99 (0.97–1.01)		1.02 (0.99–1.05)	0.99 (0.96–1.02)	
rVAT^b^, in %	0.99 (0.97–1.01)	0.98 (0.97–1.00)		1.00 (0.98–1.02)	1.00 (0.98–1.03)	

BMI, body mass index; VAT, visceral adipose tissue; SAT, subcutaneous adipose tissue; TAT, total adipose tissue; rVAT, relative visceral adipose tissue; OR, odds ratio; CI, confidence interval.

*p-value < 0.05; **p-value < 0.01; ***p-value < 0.001.

^a^Multivariable models are adjusted for age and mutually adjusted for BMI, VAT and SAT; ^b^Multivariable models are adjusted for age and BMI.

In multivariable analysis adjusted for age and BMI and mutually adjusted for other adipose tissue components (i.e. SAT for VAT and vice versa), higher VAT, TAT and rVAT were significantly associated with a reduced risk of stage IV in males only (OR 0.93; 95%CI 0.91–0.96, OR 0.95; 95%CI 0.93–0.98, OR 0.97; 95%CI 0.96–0.99, respectively). For females, all associations were in the same direction as for males but only the association for VAT was statistically significant (OR 0.95; 95%CI 0.89–1.00). No associations for SAT were found.

### Adipose tissue and Fuhrman grade

Both univariable and multivariable analyses showed no linear associations between adipose tissue parameters and Fuhrman grade in both males and females (Table 2). However, SAT higher vs. lower than the sex-specific median was associated with lower risk of high Fuhrman grade in females only (multivariable model OR 0.51; 95%CI 0.26–0.99).

### Sensitivity analyses

Supplementary Table 1 only includes patients with CT scans made within 3 months before diagnosis (N = 860, 82.8%). Results were similar to overall results in males. In females, associations of VAT with stage IV (OR 0.95; 95%CI 0.89–1.01) and of SAT higher vs. lower than the sex-specific median with Fuhrman grade (OR 0.71; 95%CI 0.36–1.39) were no longer statistically significant.

Supplementary Table 2 only includes patients with confirmed ccRCC (N = 655, 63.0%). All associations observed in males remained statistically significant. In females, associations of VAT with stage IV (OR 0.95; 95%CI 0.88–1.02) and of SAT higher vs. lower than the sex-specific median with Fuhrman grade (OR 0.57; 95%CI 0.24–1.31) were no longer statistically significant.

## Discussion

In this historical multicenter cohort study we examined associations of BMI and adipose tissue parameters with tumor stage and Fuhrman grade. We found that higher VAT, TAT and rVAT were associated with a reduced risk of stage IV compared to stage I tumors in males, after adjustment for age, BMI and SAT. Results were in the same direction in females but only the association for VAT was statistically significant. No associations were seen for BMI and SAT and no associations were found for tumor stages II and III. VAT, TAT and rVAT were not statistical significantly associated with Fuhrman grade. High SAT was associated with lower risk of high Fuhrman grade in females only.

In our study, higher VAT, TAT and rVAT were associated with a reduced risk of stage IV disease, particularly in males. This is in accordance with a Japanese study among 117 males with ccRCC which reported higher VAT in stage I patients than in stage III + IV^9^ and a Korean study in 2187 patients with stage I-IV RCC which found that patients with high vs. low VAT or rVAT had a higher proportion of stage I RCC^15^. Four studies in patients with localized RCC^10,19–21^ found no association between VAT and tumor stage. We also found no associations of adipose tissue parameters with stage II or III compared to stage I disease. In contrast, Guo et al*.* reported in 253 Chinese patients with ccRCC that patients with high rVAT had increased risk of stage III-IV compared with stage I-II disease, and this relationship was similar for males and females^8^. In a Korean study with 706 localized stage I-II RCC the highest quartile of rVAT had the highest proportion of stage I disease. However, with univariable logistic regression analysis no association of rVAT with stage was found, which is in line with our results^14^.

We found no associations between VAT, TAT and rVAT parameters and Fuhrman grade, which corresponds to one Asian and one US study in patients with localized RCC which showed no relation between VAT^19,21^ or TAT, SAT, rVAT^21^ and Fuhrman grade. A Korean study in 200 patients with stage I-a RCC found that higher VAT was significantly associated with lower ISUP grade^18^. In contrast, one US study^7^ and four Asian^10,14,16,17^ studies found that higher VAT was associated with higher Fuhrman grade. We found that high SAT was associated with lower risk of high Fuhrman grade in females only. This is in agreement with a study in RCC which reported that high rVAT (and subsequently low relative SAT) is associated with higher mortality^27^ and a study in colorectal cancer where low SAT was nonlinearly associated with higher mortality^28^. Although this may point to an antagonistic effect of SAT to VAT, neither an association for continuous SAT nor an association for rVAT was found. Therefore, this finding may as well be due to chance. Overall, we found similar results for males and females for VAT. Also for rVAT and TAT we found no strong indication for a difference between genders although results were only statistically significant for males. Only few studies investigated associations of VAT, SAT, TAT and rVAT with tumor characteristics by gender. Guo et al. reported that higher rVAT was independently associated with higher tumor stage in both males and females but found no associations for VAT, SAT and TAT^8^. On the other hand, Hu et al. reported that higher rVAT was associated with higher grade ccRCC in females but not in males^16^. Nguyen et al. found that females with higher rVAT had worse overall survival while no association for males was found^27^. It was hypothesized that this potential difference between genders might be due the sex hormone estrogen which is a key regulator of body fat distribution and is higher in females than in males and higher in females with low than those with high rVAT^8,16,27^. Activation of estrogen receptor beta by estrogen has antiproliferative effects and may reduce cell growth, migration and invasive ability in RCC tissue and increase apoptosis^29^. However, neither our nor other studies were able to include estrogen levels in the statistical analysis. Thus, whether differences in estrogen levels or other mechanisms explain the discrepancies in findings remains unclear.

Differences in findings between our and other studies may be explained by different reasons. We analysed associations of VAT, SAT, TAT and rVAT with tumor characteristics by gender while other studies did^7,17,18,21^ or did not adjust for sex^10,14,15,19,20^ or used sex-specific cut-off points^8,16^ for VAT, SAT, TAT and/or rVAT. While most studies only performed univariable analyses using means, proportions or linear regression^9,10,14,15,19,20^, we used multivariable logistic regression analysis. We investigated continuous VAT and rVAT while several other studies used medians or proportions of VAT^10,14,15,18–20^. Also, most studies were conducted in Asian populations^8–10,14–20^ which have been reported to have higher TAT and VAT compared to our Dutch population^30^, implying that results may not be directly comparable.

The mechanism underlying the relation between VAT and cancer development and progression is still to be elucidated. It is hypothesized that impaired adiponectin production and interaction between insulin and insulin-like growth factors play a key role in the development and progression of RCC^31^. However, VAT might also play a protective role as it has been demonstrated that higher VAT and SAT were associated with prolonged progression-free and overall survival^32^. Yet the mechanism behind this protective effect has not been unraveled yet^31^. It has been hypothesized that patients with obesity present themselves earlier at their physicians with other obesity-related problems^4^. They are diagnosed with less aggressive cancer subtypes at an earlier stage and therefore have a better survival^31,33^. Our results support this, given that higher VAT was associated with lower stage. Our results can also be explained by cancer cachexia^31^. Especially patients with higher stage and grade may lose significant weight, muscle mass and adipose tissue prior to RCC diagnosis due to their disease. The association between lower VAT and higher risk of stage IV disease may then be explained by reverse causality. This is supported by our finding that lower skeletal muscle index was associated with higher risk of stage IV disease (see supplementary Table 3).

This is the first large European study that examined the association of adiposity tissue parameters with TNM stage and Fuhrman grade in patients with RCC. Due to the large sample size we were able to conduct sex-specific analyses corrected for confounders and to examine stage I, II, III and IV, separately.

This study also had several limitations. First, given the observational and retrospective nature, this study was dependent on data reported in medical records and therefore susceptible to information bias and residual confounding. Potential reverse causality cannot be ruled out as a potential explanation for the association of adipose tissue parameters with tumor characteristics, as previously stated. To resolve this issue, longitudinal data is needed on patients’ initial body weight and body composition and changes in these parameters up to diagnosis^34^. However, obtaining CT scans for body composition assessment is unfeasible if there is no indication for CT imaging. Data on patient’s maximum lifetime BMI would also provide additional information and is less prone to confounding by weight loss due to disease, but was not available^34^. A sensitivity analysis including only CT scans made within 3 months before diagnosis did not alter our conclusions. Second, for 24% of the patients no suitable CT scan was available (e.g. no diagnostic scan made or a scan made at a different hospital), or CT scans had too low quality (e.g. low contrast, low signal to noise ratio resulting in a grainy appearance, or metal streak artifacts such as beam hardening or scatter effects). Patients with no CT scans were similar with respect to age and sex, but more likely to have unknown tumor stage and unknown Fuhrman grade. Third, some CT scans had incomplete cross-sectional areas of SAT (e.g. abdomen not fully visible due to poor positioning in the CT scanner). These missing SAT areas were estimated and adjusted accordingly. Last, we had many missing values for Fuhrman grade. This might be due to incomplete or missing pathology reports because some tumors (mostly with metastases) were only clinically and not pathologically confirmed. These patients were older, had lower VAT and lower TAT, had more often non-ccRCC or no specified morphology and stage 4 cancer and were not treated with surgery, implying that this was a specific group. We imputed the data accordingly to minimize the impact on our analyses.

## Conclusion

We found that higher VAT was associated with a reduced risk of stage IV RCC. This can potentially be attributed to weight loss or cancer cachexia in stage IV RCC patients only.

## Supplementary Information

Supplementary Information.

## Data Availability

The data that support the findings of this study are available from the corresponding author, A.V., upon reasonable request.

## Abbreviations

BC: Body composition
BMI: Body mass index
ccRCC: Clear cell renal cell cancer
CIs: Confidence Intervals
CT: Computed Tomography
HU: Hounsfield units
IKNL: Netherlands Comprehensive Cancer Organisation
IQR: Interquartile ranges
ISUP: International Society of Urologic Pathologists
L3: Third lumbar level
mRCC: Metastatic renal cell cancer
NCR: The Netherlands Cancer Registry
OR: Odds ratio
RCC: Renal cell cancer
rVAT: Relative visceral adipose tissue
SAT: Subcutaneous adipose tissue
SD: Standard deviations
TAT: Total adipose tissue
VAT: Visceral adipose tissue

## References

[1] World Cancer Research Fund/American Institute for Cancer Research. *Diet, Nutrition, Physical Activity and Cancer: A Global Perspective.* Continuous Update Project Expert Report 2018. dietandcancerreport.org.

[2] Kim LH (2021). A systematic review and meta-analysis of the significance of body mass index on kidney cancer outcomes. J. Urol..

[3] Balci M (2021). Differential effect of body mass index by gender on oncological outcomes in patients with renal cell carcinoma. J. Cancer Res. Ther..

[4] Parker AS (2006). Greater body mass index is associated with better pathologic features and improved outcome among patients treated surgically for clear cell renal cell carcinoma. Urology.

[5] Jeon HG (2010). Prognostic value of body mass index in Korean patients with renal cell carcinoma. J. Urol..

[6] Tsivian E (2017). Body mass index and the clinicopathological characteristics of clinically localized renal masses—An international retrospective review. Urol. Oncol..

[7] Keehn A (2015). The relationship between visceral obesity and the clinicopathologic features of patients with small renal masses. J. Endourol..

[8] Guo H (2021). The value of sex-specific abdominal visceral fat as measured via CT as a predictor of clear renal cell carcinoma T stage. Adipocyte.

[9] Naya Y (2010). Influence of visceral obesity on oncologic outcome in patients with renal cell carcinoma. Urol. Int..

[10] Zhai T, Zhang B, Qu Z, Chen C (2018). Elevated visceral obesity quantified by CT is associated with adverse postoperative outcome of laparoscopic radical nephrectomy for renal clear cell carcinoma patients. Int. Urol. Nephrol..

[11] Hakimi AA (2013). An epidemiologic and genomic investigation into the obesity paradox in renal cell carcinoma. J. Natl. Cancer Inst..

[12] Albiges L (2016). Body mass index and metastatic renal cell carcinoma: Clinical and biological correlations. J. Clin. Oncol..

[13] Crudele L, Piccinin E, Moschetta A (2021). Visceral adiposity and cancer: Role in pathogenesis and prognosis. Nutrients.

[14] Park YH (2014). Visceral obesity in predicting oncologic outcomes of localized renal cell carcinoma. J. Urol..

[15] Lee HW (2015). Prognostic significance of visceral obesity in patients with advanced renal cell carcinoma undergoing nephrectomy. Int. J. Urol..

[16] Hu Z (2020). Clear cell renal cell carcinoma: The value of sex-specific abdominal visceral fat measured on CT for prediction of Fuhrman nuclear grade. Eur. Radiol..

[17] Zhu Y (2013). Visceral obesity and risk of high grade disease in clinical t1a renal cell carcinoma. J. Urol..

[18] Park JS (2021). Association between visceral adiposity and DDX11 as a predictor of aggressiveness of small clear-cell renal-cell carcinoma: A prospective clinical trial. Cancer Metab..

[19] Kaneko G (2015). Visceral obesity is associated with better recurrence-free survival after curative surgery for Japanese patients with localized clear cell renal cell carcinoma. Jpn. J. Clin. Oncol..

[20] Hagiwara M (2012). Visceral obesity is a strong predictor of perioperative outcome in patients undergoing laparoscopic radical nephrectomy. BJU Int..

[21] Mano R (2014). Association between visceral and subcutaneous adiposity and clinicopathological outcomes in non-metastatic clear cell renal cell carcinoma. Can. Urol. Assoc. J..

[22] Bredella MA (2017). Sex differences in body composition. Adv. Exp. Med. Biol..

[23] Maurits JSF (2022). Skeletal muscle radiodensity and visceral adipose tissue index are associated with survival in renal cell cancer—A multicenter population-based cohort study. Clin. Nutr..

[24] Mourtzakis M (2008). A practical and precise approach to quantification of body composition in cancer patients using computed tomography images acquired during routine care. Appl. Physiol. Nutr. Metab..

[25] TomoVision. *SliceOmatic Alberta protocol.* http://www.tomovision.com/Sarcopenia_Help/index.htm (Aug 24, 2022; date last accessed).

[26] Lowrance WT (2010). Obesity is associated with a higher risk of clear-cell renal cell carcinoma than with other histologies. BJU Int..

[27] Nguyen GK, Mellnick VM, Yim AK, Salter A, Ippolito JE (2018). Synergy of sex differences in visceral fat measured with CT and tumor metabolism helps predict overall survival in patients with renal cell carcinoma. Radiology.

[28] van Baar H (2020). Associations of abdominal skeletal muscle mass, fat mass, and mortality among men and women with stage I-III colorectal cancer. Cancer Epidemiol. Biomark. Prev..

[29] Yu CP (2013). Estrogen inhibits renal cell carcinoma cell progression through estrogen receptor-beta activation. PLoS ONE.

[30] Wulan SN, Westerterp KR, Plasqui G (2010). Ethnic differences in body composition and the associated metabolic profile: A comparative study between Asians and Caucasians. Maturitas.

[31] Rysz J, Franczyk B, Lawinski J, Olszewski R, Gluba-Brzozka A (2020). The role of metabolic factors in renal cancers. Int. J. Mol. Sci..

[32] Steffens S (2011). Does obesity influence the prognosis of metastatic renal cell carcinoma in patients treated with vascular endothelial growth factor-targeted therapy?. Oncologist.

[33] Renfro LA (2016). Body mass index is prognostic in metastatic colorectal cancer: Pooled analysis of patients from first-line clinical trials in the ARCAD database. J. Clin. Oncol..

[34] Lennon H, Sperrin M, Badrick E, Renehan AG (2016). The obesity paradox in cancer: A review. Curr. Oncol. Rep..

