# Supplementary material for: Association of visceral and subcutaneous adiposity with tumor stage and Fuhrman grade in renal cell carcinoma
Source: Sci Rep. 2022 Oct 6;12:16718. doi: 10.1038/s41598-022-20877-2 (PMC9537539; doi:10.1038/s41598-022-20877-2)
Supplement: Supplementary file 1 — Supplementary Information. [file 41598_2022_20877_MOESM1_ESM.docx]

**Supplementary materials**


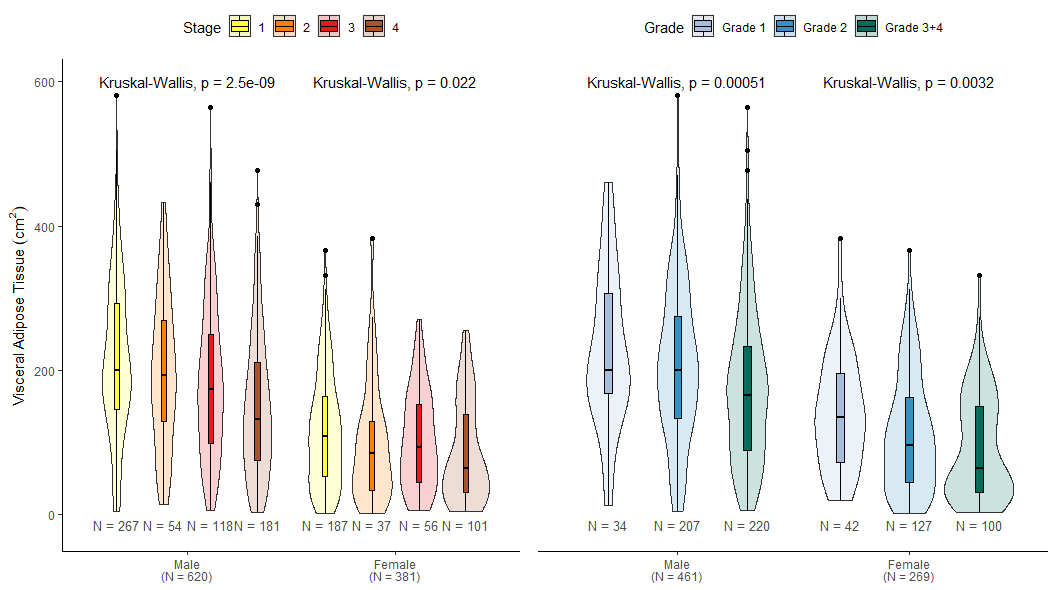


**Supplementary figure 1: Violin plots for visceral adipose tissue (cm^2^) by tumor stage and Fuhrman grade, for males and females separately. The p-values are obtained using Kruskal-Wallis tests for differences between tumor stages (stage 1 to 4) for males and females separately (left side) and between Fuhrman grades (grade 1, 2 and 3+4) for males and females separately (right side).**


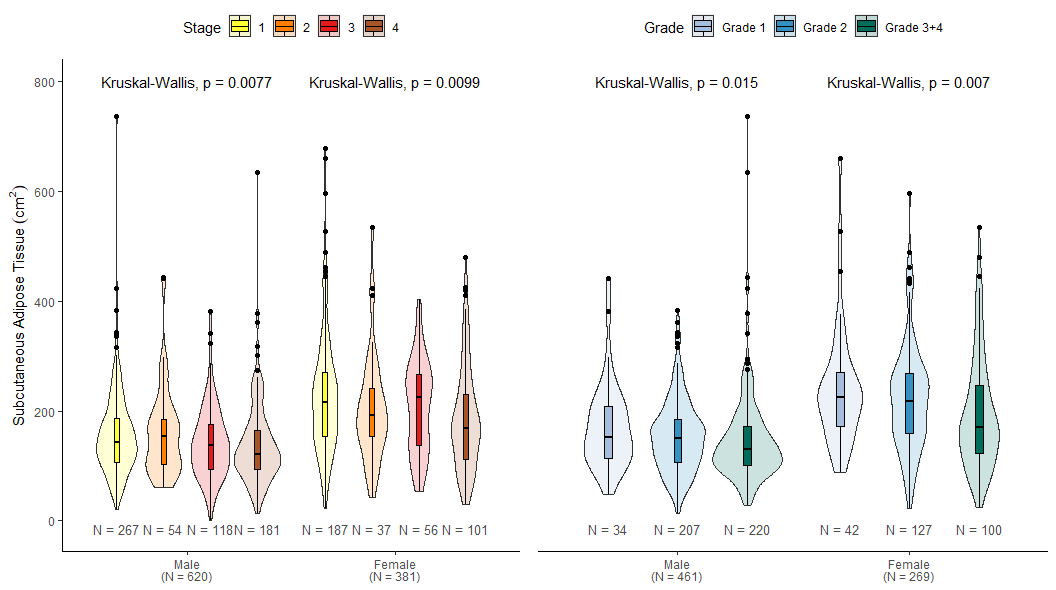


**Supplementary figure 2: Violin plots for subcutaneous adipose tissue (cm^2^) by tumor stage and Fuhrman grade, for males and females separately. The p-values are obtained using Kruskal-Wallis tests for differences between tumor stages (stage 1 to 4) for males and females separately (left side) and between Fuhrman grades (grade 1, 2 and 3+4) for males and females separately (right side).**


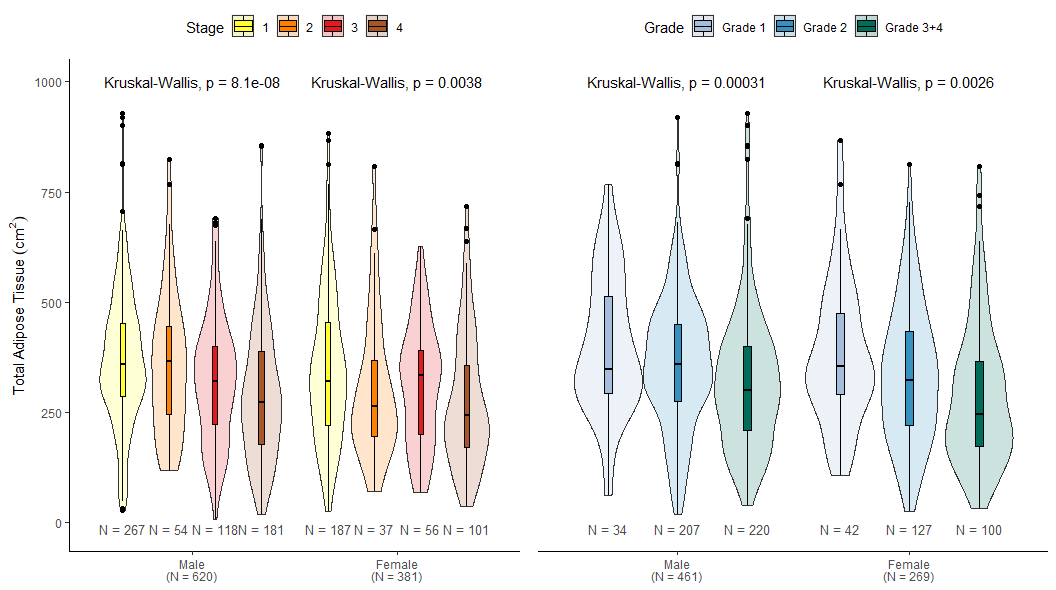


**Supplementary figure 3: Violin plots for total adipose tissue (cm^2^) by tumor stage and Fuhrman grade, for males and females separately. The p-values are obtained using Kruskal-Wallis tests for differences between tumor stages (stage 1 to 4) for males and females separately (left side) and between Fuhrman grades (grade 1, 2 and 3+4) for males and females separately (right side).**


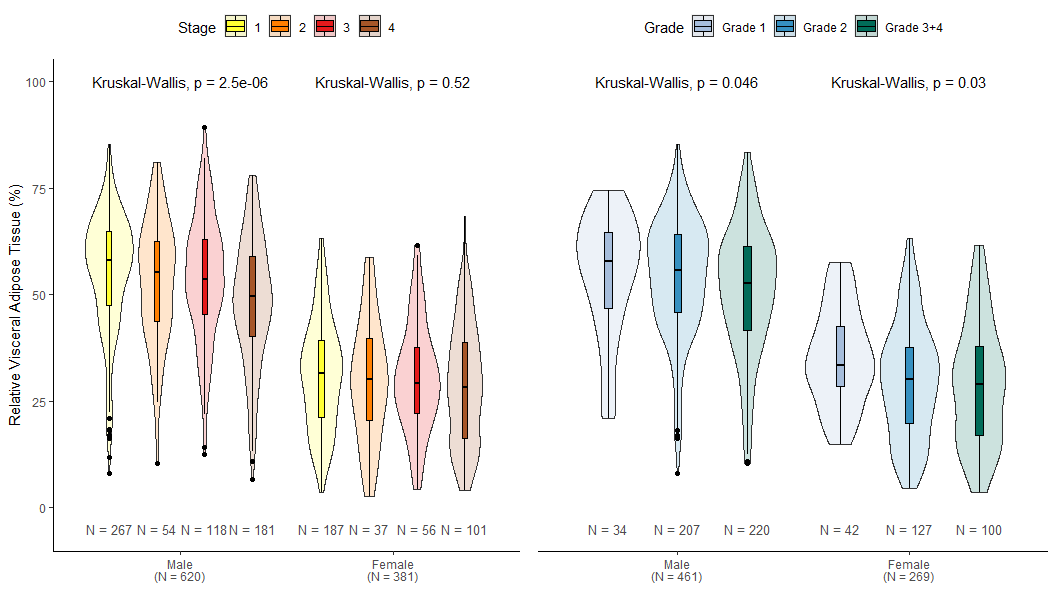


**Supplementary figure 4: Violin plots for relative visceral adipose tissue (%) by tumor stage and Fuhrman grade, for males and females separately. The p-values are obtained using Kruskal-Wallis tests for differences between tumor stages (stage 1 to 4) for males and female separately (left side) and between Fuhrman grades (grade 1, 2 and 3+4) for males and females separately (right side).**

| **Supplementary table 1: Odds ratios for body adipose tissue and TNM tumor stage (stage I as referent) and Fuhrman grade (grade 1 as referent) only in patients with CT scans within 3 months before diagnosis (N=860)** | | | | | | | | |
| --- | --- | --- | --- | --- | --- | --- | --- | --- |
| **TNM Stage**  **Stage I is referent** | **Univariable analysis** | | | | **Multivariable analysis** | | | |
|  | **Stage II**  **OR (95% CI)** | **Stage III**  **OR (95% CI)** | | **Stage IV**  **OR (95% CI)** | **Stage II**  **OR (95% CI)** | **Stage III**  **OR (95% CI)** | | **Stage IV**  **OR (95% CI)** |
|  | **Males** | | | |  |  | |  |
| BMI^1^, kg/m^2^ | 1.01 (0.94-1.09) | 0.92 (0.86-0.98)* | | 0.91 (0.86-0.97)** | 1.08 (0.94-1.25) | 0.97 (0.87-1.10) | | 1.03 (0.92-1.15) |
| VAT^1^, per 10 cm^2^ | 0.98 (0.96-1.01) | 0.97 (0.95-0.99)* | | 0.94 (0.92-0.96)*** | 0.97 (0.93-1.01) | 0.97 (0.94-1.01) | | 0.93 (0.90-0.96)*** |
| SAT^1^, per 10 cm^2^ | 1.00 (0.97-1.04) | 0.96 (0.93-1.00) | | 0.97 (0.94-1.00) | 0.99 (0.94-1.06) | 0.99 (0.94-1.05) | | 1.01 (0.96-1.06) |
| TAT^2^, per 10 cm^2^ | 0.99 (0.97-1.01) | 0.98 (0.96-0.99)** | | 0.97 (0.95-0.98)*** | 0.98 (0.94-1.01) | 0.98 (0.95-1.01) | | 0.95 (0.93-0.98)** |
| rVAT^2^, in % | 0.99 (0.97-1.01) | 0.99 (0.98-1.01) | | 0.97 (0.95-0.98)*** | 0.99 (0.97-1.01) | 0.99 (0.98-1.01) | | 0.97 (0.95-0.98)*** |
|  | **Females** | |  | |  | |  | |
| BMI^1^, kg/m^2^ | 0.94 (0.87-1.03) | 0.96 (0.90-1.03) | | 0.91 (0.86-0.98)** | 0.92 (0.78-1.08) | 0.99 (0.87-1.13) | | 0.99 (0.87-1.12) |
| VAT^1^, per 10 cm^2^ | 0.98 (0.93-1.03) | 0.98 (0.94-1.02) | | 0.95 (0.91-0.99)* | 1.02 (0.94-1.10) | 0.98 (0.92-1.05) | | 0.95 (0.89-1.01) |
| SAT^1^, per 10 cm^2^ | 0.98 (0.94-1.02) | 0.98 (0.95-1.01) | | 0.96 (0.93-0.99)** | 1.01 (0.94-1.07) | 0.99 (0.94-1.05) | | 0.98 (0.93-1.04) |
| TAT^2^, per 10 cm^2^ | 0.99 (0.96-1.01) | 0.99 (0.97-1.01) | | 0.97 (0.95-0.99)** | 1.01 (0.96-1.06) | 0.99 (0.95-1.03) | | 0.97 (0.93-1.01) |
| rVAT^2^, in % | 1.00 (0.97-1.03) | 1.00 (0.98-1.03) | | 0.99 (0.97-1.01) | 1.00 (0.97-1.04) | 0.99 (0.97-1.02) | | 0.98 (0.96-1.01) |
| **Fuhrman grade**  **Grade 1 is referent** | **Univariable analysis** | | | | **Multivariable analysis** | | | |
|  | **Grade 2**  **OR (95% CI)** | | **Grade 3+4**  **OR (95% CI)** | | **Grade 2**  **OR (95% CI)** | | **Grade 3+4**  **OR (95% CI)** | |
|  | **Males** | |  | |  | |  | |
| BMI^1^, kg/m^2^ | 1.01 (0.96-1.07) | | 0.99 (0.93-1.04) | | 0.96 (0.86-1.07) | | 0.98 (0.88-1.08) | |
| VAT^1^, per 10 cm^2^ | 1.01 (0.99-1.03) | | 0.99 (0.97-1.01) | | 1.02 (0.99-1.05) | | 0.99 (0.96-1.02) | |
| SAT^1^, per 10 cm^2^ | 1.01 (0.98-1.04) | | 1.01 (0.98-1.04) | | 1.01 (0.96-1.06) | | 1.02 (0.98-1.07) | |
| SAT^1^, high vs low | 1.56 (1.00-2.42)* | | 0.79 (0.51-1.24) | | 1.30 (0.77-2.19) | | 0.78 (0.46-1.32) | |
| TAT^2^, per 10 cm^2^ | 1.01 (0.99-1.02) | | 1.00 (0.98-1.01) | | 1.02 (0.99-1.04) | | 1.00 (0.97-1.02) | |
| rVAT^2^, in % | 1.00 (0.99-1.02) | | 0.99 (0.97-1.00) | | 1.01 (0.99-1.02) | | 0.99 (0.98-1.01) | |
|  | **Females** | |  | |  | |  | |
| BMI^1^, kg/m^2^ | 1.01 (0.96-1.06) | | 0.97 (0.92-1.03) | | 0.98 (0.88-1.08) | | 1.02 (0.91-1.15) | |
| VAT^1^, per 10 cm^2^ | 1.00 (0.96-1.03) | | 0.97 (0.93-1.00) | | 1.00 (0.95-1.06) | | 0.98 (0.93-1.04) | |
| SAT^1^, per 10 cm^2^ | 1.01 (0.99-1.04) | | 0.99 (0.96-1.01) | | 1.02 (0.98-1.06) | | 0.98 (0.94-1.03) | |
| SAT^1^, high vs low | 1.45 (0.80-2.61) | | 0.65 (0.61-1.54) | | 1.33 (0.67-2.64) | | 0.71 (0.36-1.39) | |
| TAT^2^, per 10 cm^2^ | 1.00 (0.99-1.02) | | 0.99 (0.97-1.00) | | 1.01 (0.98-1.05) | | 0.98 (0.95-1.02) | |
| rVAT^2^, in % | 0.98 (0.96-1.00) | | 0.98 (0.96-1.00) | | 0.99 (0.97-1.01) | | 1.00 (0.97-1.02) | |

Abbreviations: BMI, body mass index; VAT, visceral adipose tissue; SAT, subcutaneous adipose tissue; TAT, total adipose tissue; rVAT, relative visceral adipose tissue; OR, odds ratio; CI, confidence interval.

^1^ Multivariable models are adjusted for age and mutually adjusted for BMI, VAT and SAT; ^2^ Multivariable models are adjusted for age and BMI.

* p-value <0.05; ** p-value <0.01; *** p-value <0.001.

| **Supplementary table 2: Odds ratios for body adipose tissue and TNM tumor stage (stage I as referent) and Fuhrman grade (grade 1 as referent) in patients with ccRCC (n=655) only** | | | | | | | | |
| --- | --- | --- | --- | --- | --- | --- | --- | --- |
| **TNM Stage**  **Stage I is referent** | **Univariable analysis** | | | | **Multivariable analysis** | | | |
|  | **Stage II**  **OR (95% CI)** | **Stage III**  **OR (95% CI)** | | **Stage IV**  **OR (95% CI)** | **Stage II**  **OR (95% CI)** | **Stage III**  **OR (95% CI)** | | **Stage IV**  **OR (95% CI)** |
|  | **Males** | | | |  |  | |  |
| BMI^1^, kg/m^2^ | 1.02 (0.94-1.10) | 0.88 (0.82-0.95)** | | 0.89 (0.83-0.96)** | 1.01 (0.86-1.18) | 0.89 (0.78-1.01) | | 0.98 (0.87-1.10) |
| VAT^1^, per 10 cm^2^ | 0.99 (0.96-1.02) | 0.96 (0.93-0.98)** | | 0.94 (0.91-0.96)*** | 0.97 (0.93-1.02) | 0.96 (0.93-1.00) | | 0.93 (0.90-0.96)*** |
| SAT^1^, per 10 cm^2^ | 1.02 (0.98-1.06) | 0.97 (0.93-1.01) | | 0.97 (0.94-0.99) | 1.04 (0.97-1.11) | 1.04 (0.99-1.10) | | 1.03 (0.98-1.08) |
| TAT^2^, per 10 cm^2^ | 1.00 (0.98-1.02) | 0.97 (0.96-0.99)** | | 0.96 (0.95-0.98)*** | 0.99 (0.95-1.03) | 0.99 (0.96-1.02) | | 0.96 (0.93-0.99)** |
| rVAT^2^, in % | 0.98 (0.96-1.01) | 0.98 (0.97-1.00) | | 0.97 (0.94-0.98)*** | 0.98 (0.95-1.01) | 0.98 (0.96-1.00) | | 0.96 (0.95-0.98)*** |
|  | **Females** | |  | |  | |  | |
| BMI^1^, kg/m^2^ | 1.01 (0.92-1.10) | 0.97 (0.91-1.04) | | 0.87 (0.80-0.95)** | 1.05 (0.87-1.27) | 1.01 (0.89-1.16) | | 0.97 (0.83-1.14) |
| VAT^1^, per 10 cm^2^ | 0.99 (0.93-1.05) | 0.98 (0.94-1.03) | | 0.92 (0.88-0.97)** | 0.92 (0.82-1.02) | 0.97 (0.91-1.04) | | 0.95 (0.88-1.02) |
| SAT^1^, per 10 cm^2^ | 1.01 (0.97-1.05) | 0.98 (0.95-1.02) | | 0.94 (0.90-0.98)** | 1.04 (0.97-1.12) | 0.99 (0.94-1.05) | | 0.98 (0.92-1.04) |
| TAT^2^, per 10 cm^2^ | 1.00 (0.97-1.03) | 0.99 (0.97-1.01) | | 0.96 (0.93-0.98)*** | 0.99 (0.94-1.05) | 0.99 (0.95-1.03) | | 0.96 (0.92-1.01) |
| rVAT^2^, in % | 0.99 (0.96-1.03) | 1.00 (0.98-1.03) | | 0.98 (0.96-1.00) | 0.97 (0.92-1.01) | 0.99 (0.96-1.02) | | 0.99 (0.96-1.02) |
| **Fuhrman grade**  **Grade 1 is referent** | **Univariable analysis** | | | | **Multivariable analysis** | | | |
|  | **Grade 2**  **OR (95% CI)** | | **Grade 3+4**  **OR (95% CI)** | | **Grade 2**  **OR (95% CI)** | | **Grade 3+4**  **OR (95% CI)** | |
|  | **Males** | |  | |  | |  | |
| BMI^1^, kg/m^2^ | 1.01 (0.95-1.08) | | 0.98 (0.92-1.05) | | 0.92 (0.81-1.05) | | 0.94 (0.82-1.07) | |
| VAT^1^, per 10 cm^2^ | 1.01 (0.99-1.04) | | 0.99 (0.96-1.01) | | 1.02 (0.98-1.06) | | 0.98 (0.95-1.02) | |
| SAT^1^, per 10 cm^2^ | 1.02 (0.99-1.06) | | 1.02 (0.98-1.05) | | 1.04 (0.98-1.10) | | 1.06 (1.00-1.12) | |
| SAT^1^, high vs low | 1.86 (1.08-3.19)* | | 0.93 (0.53-1.60) | | 1.85 (0.98-3.50) | | 1.05 (0.55-1.99) | |
| TAT^2^, per 10 cm^2^ | 1.01 (0.99-1.03) | | 1.00 (0.98-1.01) | | 1.02 (0.99-1.06) | | 1.00 (0.97-1.04) | |
| rVAT^2^, in % | 1.00 (0.98-1.02) | | 0.99 (0.97-1.00) | | 1.00 (0.98-1.02) | | 0.98 (0.96-1.00) | |
|  | **Females** | |  | |  | |  | |
| BMI^1^, kg/m^2^ | 0.99 (0.93-1.05) | | 0.95 (0.89-1.02) | | 0.98 (0.87-1.11) | | 0.99 (0.87-1.14) | |
| VAT^1^, per 10 cm^2^ | 1.00 (0.96-1.04) | | 0.97 (0.93-1.01) | | 1.02 (0.95-1.08) | | 1.00 (0.93-1.07) | |
| SAT^1^, per 10 cm^2^ | 0.99 (0.97-1.02) | | 0.97 (0.94-1.01) | | 0.99 (0.94-1.04) | | 0.97 (0.92-1.03) | |
| SAT^1^, high vs low | 1.18 (0.58-2.39) | | 0.55 (0.27-1.15) | | 1.12 (0.50-2.53) | | 0.57 (0.24-1.31) | |
| TAT^2^, per 10 cm^2^ | 1.00 (0.98-1.01) | | 0.98 (0.96-1.00) | | 1.00 (0.96-1.04) | | 0.98 (0.94-1.02) | |
| rVAT^2^, in % | 0.99 (0.97-1.02) | | 0.99 (0.97-1.02) | | 1.00 (0.97-1.03) | | 1.01 (0.98-1.04) | |

Abbreviations: BMI, body mass index; VAT, visceral adipose tissue; SAT, subcutaneous adipose tissue; TAT, total adipose tissue; rVAT, relative visceral adipose tissue; OR, odds ratio; CI, confidence interval.

^1^ Multivariable models are adjusted for age and mutually adjusted for BMI, VAT and SAT; ^2^ Multivariable models are adjusted for age and BMI.

* p-value <0.05; ** p-value <0.01; *** p-value <0.001.

| **Supplementary table 3: Odds ratios for skeletal muscle index and TNM tumor stage (stage I as referent)** | | | | | | |
| --- | --- | --- | --- | --- | --- | --- |
| **TNM Stage**  **Stage I is referent** | **Univariable analysis** | | | **Multivariable analysis** | | |
|  | **Stage II**  **OR (95% CI)** | **Stage III**  **OR (95% CI)** | **Stage IV**  **OR (95% CI)** | **Stage II**  **OR (95% CI)** | **Stage III**  **OR (95% CI)** | **Stage IV**  **OR (95% CI)** |
|  | **Males** | | |  | | |
| SMI^1^, per 10 cm^2^ | 0.90 (0.63-1.31) | 0.72 (0.55-0.96)* | 0.67 (0.52-0.85)** | 0.66 (0.41-1.08) | 0.84 (0.58-1.22) | 0.65 (0.46-0.91)* |
|  | **Females** | | |  | | |
| SMI^1^, per 10 cm^2^ | 0.67 (0.37-1.23) | 0.60 (0.36-1.00) | 0.39 (0.25-0.61)*** | 0.74 (0.36-1.50) | 0.66 (0.36-1.22) | 0.48 (0.27-0.84)* |

Abbreviations: SMI, skeletal muscle index; OR, odds ratio; CI, confidence interval.

^1^ Multivariable models are adjusted for age and mutually adjusted for BMI, VAT and SAT;

* p-value <0.05; ** p-value <0.01; *** p-value <0.001.
